# LigGrep: a tool for filtering docked poses to improve virtual-screening hit rates

**DOI:** 10.1186/s13321-020-00471-2

**Published:** 2020-11-11

**Authors:** Emily J. Ha, Cara T. Lwin, Jacob D. Durrant

**Affiliations:** 1 Department of Biological Sciences, Carnegie Mellon University, Pittsburgh, PA 15213 United States; 2 Department of Biological Sciences, University of Pittsburgh, Pittsburgh, PA 15260 United States

**Keywords:** Virtual screening, Computer-aided drug discovery, Computational biology, Filters

## Abstract

Structure-based virtual screening (VS) uses computer docking to prioritize candidate small-molecule ligands for subsequent experimental testing. Docking programs evaluate molecular binding in part by predicting the geometry with which a given compound might bind a target receptor (e.g., the docked “pose” relative to a protein target). Candidate ligands predicted to participate in the same intermolecular interactions typical of known ligands (or ligands that bind related proteins) are arguably more likely to be true binders. Some docking programs allow users to apply constraints during the docking process with the goal of prioritizing these critical interactions. But these programs often have restrictive and/or expensive licenses, and many popular open-source docking programs (e.g., AutoDock Vina) lack this important functionality. We present LigGrep, a free, open-source program that addresses this limitation. As input, LigGrep accepts a protein receptor file, a directory containing many docked-compound files, and a list of user-specified filters describing critical receptor/ligand interactions. LigGrep evaluates each docked pose and outputs the names of the compounds with poses that pass all filters. To demonstrate utility, we show that LigGrep can improve the hit rates of test VS targeting *H. sapiens* poly(ADPribose) polymerase 1 (*Hs*PARP1), *H. sapiens* peptidyl-prolyl cis-trans isomerase NIMA-interacting 1 (*Hs*Pin1p), and *S. cerevisiae* hexokinase-2 (*Sc*Hxk2p). We hope that LigGrep will be a useful tool for the computational biology community. A copy is available free of charge at http://durrantlab.com/liggrep/.

## Introduction

Traditional high-throughput screening (HTS) is a powerful experimental technique for identifying molecules that can be further developed into novel pharmaceutics. It involves screening a large number of compounds against selected disease targets (e.g., disease-relevant proteins) to find compounds (i.e., “hits”) that elicit measurable biological or biophysical responses. HTS does not require any prior knowledge of the drug-target structure. But the associated hit rates are often low, and it is costly to experimentally test so many molecules. HTS is thus limited primarily to research groups working in the pharmaceutical industry [1].

Computer-aided drug discovery (CADD) aims to address some of these challenges. Among CADD techniques, structure-based virtual screening (VS) is particularly popular. VS draws on a library of virtual small molecules and a model of the drug target. A docking program first predicts how each molecule physically interacts with the target protein, often by binding to a pocket on the target surface [2]. The geometry of the bound molecule relative to the target structure is called the docked pose. Based on this pose, the docking program then estimates the strength of binding (the docking score) using a pre-determined scoring function [3]. The set of compounds with reasonable docked poses and good docking scores is often enriched with true binders. These candidate ligands are then prioritized for subsequent experimental testing [1, 4]. This targeted-search approach reduces cost, preparation time, and workload relative to HTS.

Assessing candidate ligands by docking score is straightforward, but assessing predicted poses is often more subjective and time consuming. One objective pose-assessment approach is to search for new molecules that are predicted to participate in the same critical interactions with the drug target that are typical of known ligands. For example, all clinically approved inhibitors of influenza neuraminidase interact with certain key arginine residues [5], so docked compounds that are not predicted to participate in those interactions might be discarded regardless of score. Unfortunately, in the case of large-scale VS there may be many thousands (or even millions) of docked poses, complicating comprehensive manual inspection.

To automate pose assessment, some commercial docking programs (e.g., Schrödinger’s Glide and OpenEye’s FRED) automatically reject poses that do not satisfy user-specified filters. These programs are powerful, but they have restrictive and/or expensive licenses that make them inaccessible to many groups. Even OpenEye’s free academic license imposes substantial commercialization and intellectual-property restrictions. In contrast, many popular open-source docking programs (e.g., AutoDock Vina [6]) have far more permissive licenses, but they often lack a pose-filtering step.

To address this limitation, we have created LigGrep, a free, open-source program that filters predicted ligand poses that were previously generated during a VS campaign. LigGrep analyzes the pose of each docked compound and discards those poses that do not participate in user-specified interactions. Alternatively, it can also discard poses that involve unfavorable interactions (e.g., interactions with residues known to be involved in resistance mechanisms). LigGrep will help improve VS hit rates by allowing the community to focus on high-scoring compounds that also satisfy carefully chosen binding-pose criteria. A copy is available at http://durrantlab.com/liggrep/, released under the terms of the Apache License, Version 2.0.

## Implementation

### The LigGrep algorithm

LigGrep accepts as input (1) a PDBQT or PDB file of the drug-target receptor used for docking, (2) a directory of PDBQT, PDB, or SDF files containing the docked poses of candidate ligands, and (3) a JSON-formatted file describing user-specified filters. After evaluating each docked pose, it outputs the names of any compounds with poses that satisfy all user-defined filters. The following sections describe each of these steps in detail.

#### Input receptor molecule

LigGrep’s first command-line argument is the path to the PDB/PDBQT-formatted receptor file used for docking. LigGrep uses the Scoria Python library [7] to load the receptor file. Because Scoria is a pure-Python library with a permissive license, we package a copy together with LigGrep itself for convenience.

#### Input ligand molecules

LigGrep’s second command-line argument is the path to a directory containing the docked-compound files. To accommodate a broad range of docking programs, we designed LigGrep to accept docked poses in three popular file formats. These include the PDBQT format to accommodate docking programs such as AutoDock Vina [6], the PDB format to accommodate big-data studies of crystallographic poses, and the SDF format to accommodate programs such as Schrödinger’s Glide. Researchers who wish to examine predicted or experimentally determined poses saved in other formats can convert to the SDF format using open-source programs such as Open Babel [8].

In many cases, users will wish to filter poses by considering only a single ligand atom (e.g., “which poses place an oxygen atom in a particular region of the binding pocket?”). In other cases, users may wish to filter poses using more complex ligand substructures (e.g., “which poses place a hydroxyl group in a particular region?”). In still other cases, substructures with specific atomic-bond specifications may be critical (e.g., “which poses place a phenyl group–but not a cyclohexyl group–in a particular region?”). Users can run LigGrep in three modes (NONE, OPENBABEL, and SMILES) according to their needs.

**NONE mode**. In NONE mode (*--mode NONE*), LigGrep does not assign bond orders beyond those described in the docked-compound files themselves. In the case of PDB/PDBQT files, which do not include any information about bond orders, LigGrep assumes that all atoms are sp^3^ hybridized and that all appropriately juxtaposed atoms are connected by single bonds. In the case of SDF files, which do include detailed information about atomic bonds, LigGrep instead assigns bond orders based on the information present in the SDF files themselves. NONE mode is thus ideal when (1) filtering by a single atom, (2) filtering by a substructure whose atoms are connected only by single bonds, or (3) applying filters of any type to SDF-formatted docked poses.

**SMILES mode**. In SMILES mode (*--mode SMILES*), LigGrep assigns bond orders to PDB/PDBQT-formatted docked-compound files by additionally considering user-specified files in the SMILES format. The simplified molecular-input line-entry system (SMILES) format is a widely used, compact format that encodes a molecule’s connectivity and chirality as a simple, one-line string of letters and other symbols. To run LigGrep in SMILES mode, the user should save a separate SMILES file for each PDB/PDBQT-formatted docked compound, in the same compound directory. Each SMILES file should have the same filename as the corresponding PDB/PDBQT file, plus the “.smi” extension. SMILES mode is ideal when (1) SMILES strings are available or can be easily generated, (2) filters involve substructures with higher-order (e.g., double, triple, aromatic) bonds, and (3) the docked files are PDB/PDBQT formatted. SMILES mode is not appropriate for SDF-formatted files because these already include bond-order information.

**OPENBABEL mode**.  In OPENBABEL mode (*--mode OPENBABEL*), LigGrep uses the Open Babel (*obabel*) executable [8] to try to assign atom hybridization and bond orders to docked-compound PDB/PDBQT files. Users must specify the path to the Open Babel executable using LigGrep’s *--babel_exec /PATH/TO/OBABEL* parameter. Internally, Open Babel converts PDB/PDBQT files to the SDF format, which includes bond-order information that the Python library RDKit [9] can then process. Unfortunately, PDBQT files do not include non-polar hydrogen atoms, complicating this process. LigGrep runs Open Babel first using the *−h* option to add all missing hydrogen atoms. If that hydrogen-atom assignment does not match the atoms present in the input PDB/PDBQT file, LigGrep reruns Open Babel using the *−p* option, which adds hydrogen atoms as appropriate for neutral pH (7.4). If this second attempt fails, LigGrep issues a warning and moves on to the next docked-compound file.

We provide OPENBABEL mode for user convenience. It allows users to process PDB/PDBQT-formatted poses using higher-order substructure filters, even when SMILES strings are not available. But we recommend using OPENBABEL mode cautiously. Given the ambiguities associated with assigning hybridization and bond orders based on atomic coordinates alone, LigGrep in OPENBABEL mode may misclassify some compounds. Furthermore, OPENBABEL mode is not appropriate when filtering SDF-formatted poses because SDF files already include bond-order information.

#### Input-filters file

**Fig. 1:**
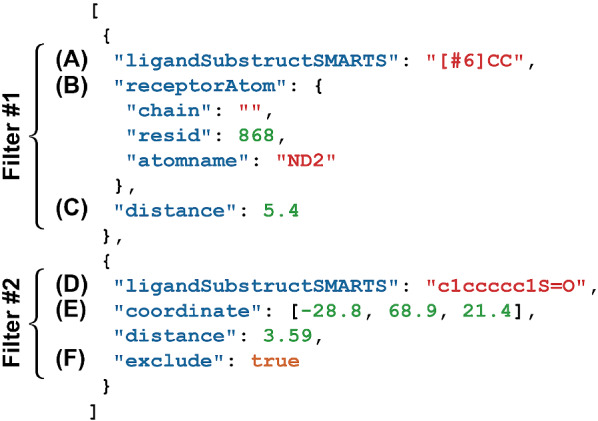
Sample JSON file containing hypothetical filters. Filter #1 shows a 3D query point specified by a receptor atom. Filter #2 shows a 3D query point specified by a coordinate, with the exclude flag set

LigGrep’s third command-line argument is the path to a JSON file containing a list of filters that the input compounds must satisfy (Fig. 1). LigGrep filters have four user-defined components: (1) a ligand-substructure specification describing one or more bonded atoms, (2) a point in 3D space (the query point), (3) a distance cutoff, and (4) an optional “exclude” flag.

**Identifying ligand substructures**. To determine whether a given docked pose satisfies the user-specified filters list, LigGrep first uses the RDKit Python library [9] to check whether the molecule contains the necessary substructures (i.e., the substructures associated with all filters that do not have “exclude” flags, Fig. 1a). LigGrep rejects all molecules that do not contain each of the necessary substructures. Users specify substructures via SMILES arbitrary target specification (SMARTS) notation (Fig. 1a and d), which is syntactically similar to SMILES. When using LigGrep in *NONE* mode to filter PDB/PDBQT-formatted docked molecules, substructures must include only sp^3^-hybridized atoms connected by single bonds (as in Fig. 1a). Otherwise, the SMARTS strings can include more complex substructure descriptions (e.g., aromatic rings, as in Fig. 1d).

**Identifying the 3D query point**. For each specified filter, LigGrep identifies the appropriate 3D query point by examining the corresponding JSON data. If the filter JSON contains the key “coordinate”, LigGrep constructs the 3D query point directly from the corresponding value, a list containing x, y, and z coordinates (Fig. 1e). If the filter JSON contains the key “receptorAtom”, LigGrep constructs the 3D query point by searching the input receptor file for an atom that matches the provided chain, residue id (resid), and atom name (atomname) (Fig. 1b). The 3D query point is then set to the coordinate of that atom.

**Accepting or rejecting docked compounds based on atomic distances**. Once LigGrep has identified the relevant small-molecule substructures and 3D query points, it determines whether a given docked pose passes or fails each filter. If it passes all filters, the name of the compound file is saved to an output text file. If it fails any of the filters, the compound file name is not saved. The filters are thus combined using a Boolean AND (conjunction) operator, though advanced users familiar with SMARTS notation can also embed additional logical operators within their substructure specifications for more complex, nested logic.

To apply each filter, LigGrep first calculates the minimum distance between any small-molecule-substructure atom and the corresponding 3D query point. It then compares this calculated distance to the cutoff distance associated with each filter (Fig. 1c). By default, a given docked pose passes a filter if it positions the substructure near the query point. If the user includes an optional “exclude” flag (Fig. 1f), a pose passes only if it does not position the substructure near the query point. The specific criteria used to assess each filter are given in Table 1.

**Table 1:** LigGrep criteria for assessing user-defined filters

Has substructure	Exclude flag set	Any substructure atom within cutoff distance	Result
No	No	N/a	Fail
No	Yes	N/a	Pass
Yes	No	No	Fail
Yes	No	Yes	Pass
Yes	Yes	No	Pass
Yes	Yes	Yes	Fail

### Dependencies and compatibility

We have tested LigGrep on macOS, Ubuntu Linux, and Windows 10 (Table 2). LigGrep requires the third-party Python libraries RDKit [9], NumPy [10], and SciPy [11], which must be installed separately. It comes pre-packaged with the Scoria library [7] for convenience. When running LigGrep in the preferred NONE or SMILES mode, the Open Babel executable is not required. Users who wish to use OPENBABEL mode must install Open Babel separately.

**Table 2:** We have tested LigGrep on various operating systems, using various versions of Python 3, RDKit, NumPy, SciPy, and Open Babel

Operating system	Python	RDKit	NumPy	SciPy	Open babel	Multiprocessing
macOS Mojave 10.14.5	3.6.7	2019.03.3	1.17.4	1.3.1	2.4.1	Y
Ubuntu 18.04.4 LTS	3.6.5	2019.03.1	1.16.3	1.3.0	3.1.0	Y
Ubuntu 18.04.2 LTS	3.7.3	2020.03.3	1.18.1	1.4.1	2.3.2	Y
Windows 10 Home 1903	3.7.4	2020.03.2	1.16.5	1.3.1	2.3.1	N

Multiprocessing is not supported on Windows. Note that LigGrep is designed to work with Python 3, not Python 2

### Benchmark virtual screens

#### Preparing the receptors

To demonstrate LigGrep use, we performed VS against three proteins: *H. sapiens* poly(ADP-ribose) polymerase 1 (*Hs*PARP1), *H. sapiens* peptidyl-prolyl cis-trans isomerase NIMA-interacting 1 (*Hs*Pin1p), and *S. cerevisiae* hexokinase-2 (*Sc*Hxk2p). We downloaded *Hs*PARP1, *Hs*Pin1p, and *Sc*Hxk2p crystal structures (6BHV [12], 3TDB [13], and 1IG8 [14], respectively) from the RCSB Protein Data Bank [15, 16]. We selected the 6BHV *Hs*PARP1 structure because its co-crystallized ligand, benzamide adenine nucleotide, was the largest by mass of 46 co-crystallized ligands considered (Additional file 1: Table S1), and some studies suggest that larger binding-pocket conformations are more amenable to VS [17, 18]. We selected the 3TDB *Hs*Pin1p structure because its co-crystallized ligand was the largest by mass of 27 considered (Additional file 1: Table S2). Finally, we selected the 1IG8 *Sc*Hxk2p structure because it is the only *Sc*Hxk2p structure with the correct amino-acid sequence. In all cases, we used the PDB2PQR server [19–21] to add hydrogen atoms to these protein structures and to optimize their hydrogen-bond networks (default parameters, pH 7). We then used Open Babel [8] to convert the resulting protonated PQR files to the PDB format. Finally, we used MGLTools 1.5.6 [22] to convert the PDB files to the PDBQT format, which includes atom types and partial atomic charges.

#### Preparing the small-molecule libraries

To prepare a library of small molecules for *Hs*PARP1 docking, we downloaded the SMILES strings of 46 known *Hs*PARP1 ligands (Additional file 1: Table S1) and 1515 diverse small molecules (presumed decoys). The known ligands were taken from *Hs*PARP1 crystal structures deposited in the RCSB Protein Data Bank, and the decoys were taken from the NCI Diversity Set VI, a set of freely available compounds provided by the National Cancer Institute (NCI) [23]. We created a similar small-molecule library of known ligands and NCI decoys for *Hs*Pin1p docking, using 27 co-crystallized *Hs*Pin1p ligands (Additional file 1: Table S2). For *Sc*Hxk2p docking, we identified 41 glucose analogues known to bind hexokinase and glucokinase proteins from various species (presumed *Sc*Hxk2p ligands; eight from the RCSB Protein Data Bank, and 33 from the BindingDB database [24, 25]; Additional file 1: Table S3). As *Sc*Hxk2p decoys, we selected 1652 glucose analogues with molecular weights less than 500 Daltons, taken from the ChemDiv and eMolecules databases (presumed inactives).

We used the open-source program Gypsum-DL [4] to generate 3D small-molecule models from these SMILES strings. To account for alternate ionization, tautomeric, chiral, isomeric, and ring-conformational forms, we instructed Gypsum-DL to generate two molecular variants per input compound (*min_ph*: 7.4; *max_ph*: 7.4; *pka_precision*: 0). We also used Gypsum-DL’s *--use_durrant_lab_filters* flag to remove molecular variants judged improbable. The output small-molecule PDB files were again converted to the PDBQT format using MGLTools 1.5.6 [22].

#### Docking

To prepare for docking, we used AutoDockTools [22] and Webina [26] to identify a docking box centered on the ligand-binding pockets of the respective crystal structures. In the case of the 6BHV *Hs*PARP1 structure, we retained all four catalytic domains present in the 6BHV asymmetric unit for simplicity’s sake, but the docking box (23 Å x 17 Å x 20 Å) encompassed only chain-A atoms. The *Hs*Pin1p and *Sc*Hxk2p docking boxes were 20 Å x 20 Å x 20 Å and 20 Å x 19 Å x 15 Å, respectively. We docked all compounds into their respective receptors using AutoDock Vina [6], with Vina’s default parameters.

#### Defining LigGrep filters

To construct LigGrep filters suitable for the HsPARP1 VS,PARP1 VS, we reviewed a recently published table of predicted *Hs*PARP1/ligand interactions that were frequently identified in a large-scale de novo CADD campaign [27]. Two of the most frequent interactions were also observed in crystal structures of *Hs*PARP1 bound to the clinically approved inhibitor niraparib (4R6E:A [28]) and a 4(3*H*)-quinazolinone derivative (1UK0:A [29]): a hydrogen bond with G863, and a $\pi$-$\pi$ interaction with Y907. Based on our analysis of these structures, we defined two structural (“receptorAtom”) filters to capture the two interactions. To capture the hydrogen bond, we required that a docked-compound nitrogen or oxygen atom (SMARTS: *[#7,#8]*) come within 5.5 Å of the G863 alpha carbon (*resid*: 863; *atomname*: CA). To capture the $\pi$-$\pi$ interaction, we required that a docked-compound carbon-carbon aromatic bond (SMARTS: *cc*) come within 5.5 Å of the most distal Y907 carbon atom (*resid*: 907; *atomname*: CZ).

To construct filters suitable for the *Hs*PARP1 VS, we examined multiple co-crystallized ligands deposited in the RCSB Protein Data Bank. Many of these positioned peptide-backbone-like substructures (e.g., imidazole- and furan-carboxylic-acid moieties) near where endogenous peptides bind [30]. We therefore defined one “coordinate” filter to identify docked poses that positioned *[N,O]CCO* substructures within 4.0 Å of this position. We used a SMARTS string with only sp^3^-hybridized atoms (single bonds) to ensure compatibility with LigGrep’s NONE mode.

To construct filters suitable for the *Hs*Pin1p VS, we again considered known ligands. Many hexokinase ligands are glucose analogues, so we defined a single “coordinate” filter to identify docked poses that positioned a tetrahydro-2*H*-pyran moiety (*C1OCCCC1*) within 1.0 Å of the 3D position where glucose typically binds.

#### Measuring virtual screen performance

To evaluate the performance of our VS in terms of both the docked poses and the associated docking scores, we first selected a single candidate pose for each unique input molecule in our library. Given that Gypsum-DL generated up to two molecular variants for each molecule and that Vina predicted up to 9 poses for each variant, each unique molecule was associated with at most 18 poses. In practice this number was smaller on average because in some cases Gypsum-DL generated only one variant, and LigGrep (when applied) filtered out those poses that failed to meet our user-specified criteria. To assess the VS before applying LigGrep, we considered only each ligand’s single, top-scoring pose, without regard for pose geometry. To assess the VS after applying LigGrep, we considered the top-scoring pose from among the poses with geometries that matched our user-defined LigGrep filters.

**Pose accuracy**. To assess how well Vina predicts the poses of known *Hs*PARP1 ligands, we used UCSF Chimera [31] to align the 46 *apo* *Hs*PARP1 crystal structures listed in Additional file 1: Table S1 to the *Hs*PARP1 structure used for docking. We then used the *obrms* program, included in the Open Babel package [8], to calculate the root-mean-square deviation (RMSD) between the top-scoring docked pose of each known ligand and the corresponding crystallographic pose (Additional file 1: Table S1). We used this same protocol to assess how well Vina predicts the poses of known *Hs*Pin1p and *Sc*Hxk2p ligands (Additional file 1: Tables S2 and S3), though in the case of *Sc*Hxk2p only one crystallographic ligand was available.

**Scoring (ranking) accuracy**. We used several methods to assess how well the three VS ranked known ligands above decoy molecules, both before and after applying LigGrep. First, we ordered the compounds of the three VS by their docking scores and calculated the percentile ranks of the known ligands (Additional file 1: Tables S1–S3). Second, we counted the number of positive-control compounds that ranked in the top 10, 20, and 40 compounds for each VS (Table 3). Third, we calculated enrichment factors. Given a VS of *T* total small molecules including $P_{T}$ positive-control compounds, the enrichment factor, $EF_{n}$, of the *n* top-ranked compounds is the number of positive-control ligands present, $P_{n}$, divided by the number of positive controls that would be expected if the compounds were randomly ordered, i.e., $EF_{n} = P_{n} / (nP_{T} / T)$. Finally, though LigGrep is best used to enrich the set of top-ranked compounds, we also assessed the impact of LigGrep filters on the entire set of ranked compounds using receiver operating characteristic (ROC) metrics (see Additional file 1: Figure S1).

## Results and discussion

### Benchmark virtual screen: *Hs*PARP1

To demonstrate utility, we first used LigGrep to enrich a VS targeting the *Hs*PARP1 catalytic pocket. *Hs*PARP1 is a highly conserved, multifunctional enzyme that plays important roles in the DNA-damage response. It post-transcriptionally attaches a negatively charged polymer termed poly(ADP-ribose) (PAR) to various protein targets (including itself). These PAR chains recruit proteins that contribute to DNA repair, to the stabilization of DNA replication forks, and to the modification of chromatin structure [32]. *Hs*PARP1 is over-expressed in various carcinomas, making it a potential therapeutic target. Additionally, multiple preclinical research studies and clinical trials demonstrate that *Hs*PARP1 inhibition can repress tumor growth and metastasis [33].

Although a few *Hs*PARP1 inhibitors have been approved for clinical use (e.g., olaparib, niraparib, and rucaparib) [34], clinical trials have revealed a number of therapeutic limitations. These limitations include (1) toxicity due to promiscuous binding, given that most *Hs*PARP1 inhibitors resemble NAD+ [35]; (2) activation of viral replication, especially the replication human T-cell lymphotropic virus (HTLV) or Kaposi’s sarcoma-associated herpes virus (KSHV) [35]; and (3) acquired resistance that limits long-term use [36]. There is thus an urgent need for novel ligands that can be further developed into clinically useful *Hs*PARP1 inhibitors.

#### Unfiltered *Hs*PARP1 virtual screen

We first performed a standard VS on *Hs*PARP1 to establish baseline performance. The screen involved 46 co-crystallized (known) *Hs*PARP1 catalytic inhibitors, as well as 1515 additional molecules that served as decoys (presumed inactives). We evaluated our Vina-based docking protocol both in terms of pose-prediction accuracy and the ability to distinguish between known inhibitors and decoy molecules. To evaluate the accuracy of the predicted poses, we calculated heavy-atom RMSDs between the top-scoring docked and crystallographic poses of the 46 known inhibitors included in our small-molecule library. The average RMSD value was 2.82 Å (± 2.04 Å stdev), ranging from 0.48 Å (4OPX:A) to 12.14 Å (4HHY:A). Thirty-one of the known *Hs*PARP1 inhibitors had top-scoring docked poses within 3 Å of the crystallographic pose (Additional file 1: Table S1). These RMSD calculations indicate that Vina is reasonably adept at posing the known ligands correctly.

To evaluate how well the Vina-based docking protocol can distinguish between known inhibitors and decoy molecules, we ranked the compounds of our small-molecule library by the docking scores of their top-scoring poses. We found that 45 of 46 known catalytic inhibitors ranked in the top 40%, and 28 ranked in the top 10%. The single highest-scoring compound was in fact a known *Hs*PARP1 catalytic inhibitor (“compound 33” from the 4HHY structure [37]); five known ligands were in the top 10 compounds, seven in the top 20, and 13 in the top 40 (Table 3 and Additional file 1: Table S1).

**Table 3:** The number of known ligands caught in the top 10, 20, and 40 highest-scoring compounds of each VS when docking with Vina only vs. Vina + LigGrep

Protein	Screen type	Top 10	Top 20	Top 40
*Hs*PARP1	Vina only	5/46	7/46	13/46
*Hs*PARP1	Vina + LigGrep	7/46	11/46	19/46
*Hs*Pin1p	Vina only	2/27	3/27	5/27
*Hs*Pin1p	Vina + LigGrep	3/27	4/27	8/27
*Sc*Hxk2p	Vina only	2/41	4/41	6/41
*Sc*Hxk2p	Vina + LigGrep	4/41	6/41	7/41

#### LigGrep-filtered *Hs*PARP1 virtual screen

To show how LigGrep can further improve the hit rate of this high-performing VS, we filtered the docked poses of all library compounds to identify those predicted to participate in a hydrogen bond with the *Hs*PARP1 G863 residue and a $\pi$-$\pi$ interaction with Y907 (Table 3, see “Implementation” for details). LigGrep filtered out 435 of the 1561 unique compounds (ligands + decoys) in the virtual library. We ranked (by docking score) the remaining 1126 unique compounds with poses that matched our filter criteria.

Importantly, LigGrep allowed us to effectively consider all the poses associated with each docked compound, not just the top-scoring pose. Given our Gypsum-DL/Vina protocol (see “Implementation”) [4, 6], each unique compound in our library was associated with up to 18 predicted poses. Manually inspecting so many poses is impossible. Our approach in the past has been to consider only the top-scoring pose associated with each Vina run, and then to visually inspect only the poses of the top-ranked docked compounds. LigGrep now allows us to consider all docked poses associated with each candidate ligand, not just the top-scoring pose.

**Fig. 2:**
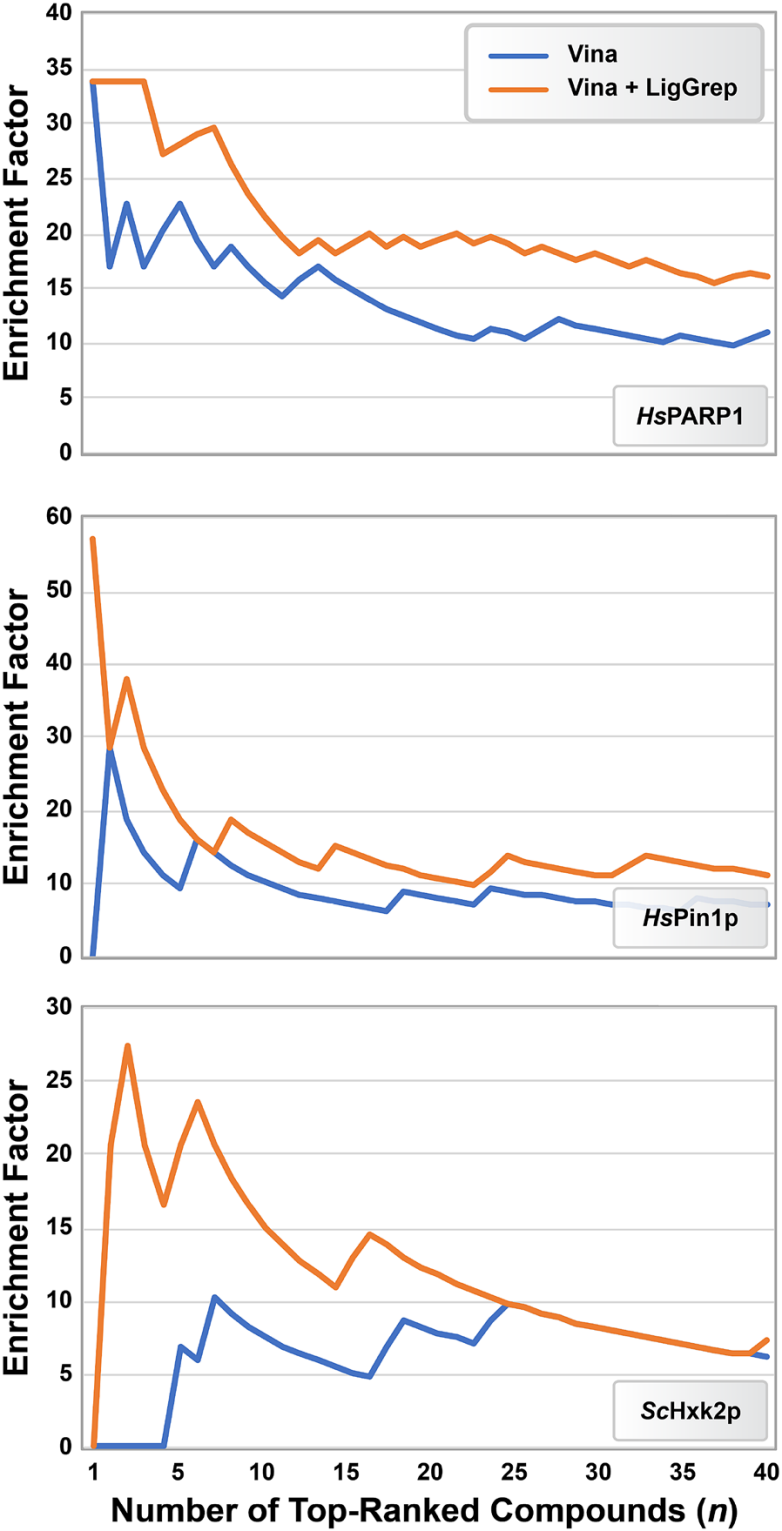
Enrichment factors associated with our *Hs*PARP1,*Hs*Pin1p, and *Sc*Hxk2p VS, before (blue) and after (orange) applying LigGrep filters. To calculate the enrichment factors of the LigGrep-filtered VS, any compound that did not pass the filter(s), whether a positive control or decoy, was moved to the bottom of the ranked list

After we used LigGrep to filter out less-likely poses, seven known ligands ranked in the top 10 highest-scoring compounds, 11 in the top 20, and 19 in the top 40 (Table 3 and Additional file 1: Table S1). In fact the top four ranked compounds were all known *Hs*PARP1 inhibitors. LigGrep thus improved the hit rate among the top-scoring compounds over that obtained using the unfiltered VS. Similar improvements can be seen in enrichment factors (Fig. 2) and areas under the pROC curve (Additional file 1: Figure S1 and Table S4).

### Benchmark virtual screen: *Hs*Pin1p

To further demonstrate utility, we also applied LigGrep to a VS targeting *Hs*Pin1p. *Hs*Pin1p binds to proteins with phosphorylated serine/threonine-proline (pSer/Thr-Pro) motifs and accelerates the cis–trans isomerization of the proline residue. Peptidyl-prolyl isomerization can be seen as a kind of post-translational modification that impacts how many proteins fold, localize, activate, and interact [38–41]. *Hs*Pin1p is upregulated in many cancers, likely because many of its targets contribute to cancer pathogenesis. Reduced *Hs*Pin1p activity protects against cancer progression [38, 42–44], so much effort has been invested in developing *Hs*Pin1p inhibitors [38].

#### Unfiltered *Hs*Pin1p virtual screen

As with *Hs*PARP1, we first performed a standard *Hs*Pin1p VS to establish baseline performance. The screen involved 27 co-crystallized (known) *Hs*Pin1p inhibitors, as well as 1515 additional molecules that served as decoys (presumed inactives). The average heavy-atom RMSD between the top-scoring docked and crystallographic poses of the 27 known inhibitors was 3.43 Å (± 2.33 Å stdev), ranging from 0.26 Å (2XP6:A) to 8.63 Å (3TDB:A). Fourteen of the known *Hs*Pin1p inhibitors had top-scoring docked poses within 3 Å of the crystallographic pose (Additional file 1: Table S2).

We again ranked the compounds of our small-molecule library by the docking scores of their top-scoring poses. We found that 23 of 27 known inhibitors ranked in the top 40%, and 12 ranked in the top 10%. Two known ligands were in the top 10 compounds, three in the top 20, and five in the top 40 (Table 3 and Additional file 1: Table S2).

#### LigGrep-filtered *Hs*Pin1p virtual screen

To show how LigGrep can further improve the hit rate of the *Hs*Pin1p VS, we filtered the docked poses of all library compounds to identify those that positioned peptide-backbone-like substructures near where endogenous peptides bind (Table 3, see “Implementation” for details) [30]. Given that this substructure is very specific, LigGrep filtered out 1029 of the 1542 unique ligands and decoys. We ranked (by docking score) the remaining 513 unique compounds with poses that matched our filter criteria, including 25 of the known ligands.

After LigGrep filtering, three known ligands ranked in the top 10 highest-scoring compounds, four in the top 20, and eight in the top 40 (Table 3 and Additional file 1: Table S2). LigGrep thus improved the hit rate among the top-scoring compounds. In fact the top-ranked compound after LigGrep filtering was the known *Hs*Pin1p inhibitor 3-(6-fluoro-1H-benzimidazol-2-yl)-N-(naphthalen-2-ylcarbonyl)-D-alanine, with a measured K_i_ of 80 nM [45]. The enrichment factors (Fig. 2) and areas under the pROC curve similarly improved (Additional file 1: Figure S1 and Table S4).

### Benchmark virtual screen: *Sc*Hxk2p

As a final demonstration, we applied LigGrep to a VS targeting *Sc*Hxk2p. Hexokinases perform the first step in glucose metabolism, transferring an ATP $\gamma$ phosphate to a glucose C6 carbon atom to produce glucose-6-phosphate (Glc-6P) [46]. This activated Glc-6P is critical for downstream catabolic processes such as anaerobic fermentation [47] and aerobic oxidative phosphorylation (OXPHOS) [47], as well as anabolic pathways such as the pentose–phosphate shunt [48–50].

Glucose metabolism is often dysregulated in cancer. Cancer cells tend to use glycolysis and lactic acid fermentation to generate ATP from glucose, even when adequate oxygen is available for the more efficient OXPHOS [49, 51–55]. To maintain ATP levels, cancer cells must increase glycolytic flux [50], often by upregulating hexokinase II (*Hs*HK2p) [48, 49, 56, 57]. Several groups have developed small-molecule *Hs*HK2p ligands that bind to the catalytic pocket [48, 58–63], but acquired resistance [64–71] and unacceptable toxicities [72] require the development of additional hexokinase inhibitors.

#### Unfiltered *Sc*Hxk2p virtual screen

We first performed a standard *Sc*Hxk2p VS to establish baseline performance. We designed this VS to determine whether LigGrep can enhance performance even in challenging circumstances. First, rather than limit our set of positive controls (“known ligands”) to *Sc*Hxk2p ligands, we selected 41 glucose analogues known to bind hexokinases and glucokinases from any of several species. It is therefore likely that some of our “positive controls” are not true *Sc*Hxk2p ligands, effectively injecting noise into our VS signal. To further exacerbate this challenge, we selected molecules that are chemically similar to the positive controls to serve as presumed inactive decoys (i.e., 1652 glucose analogues present in the ChemDiv and eMolecules databases).

Second, we of necessity had to limit our pre-VS assessment of pose accuracy. Only one of the selected positive-control compounds (ortho-toluoylglucosamine [73]) has been co-crystallized with *Sc*Hxk2p. The heavy-atom RMSD between the top-scoring and crystallographic poses of this compound was 1.90 Å, suggesting that Vina is well suited to *Sc*Hxk2p docking, but the available structures permit only this one data point as validation.

We again ranked the compounds of our small-molecule library by the docking scores of their top-scoring poses. We found that 26 of 41 positive-control compounds ranked in the top 40%, and 12 ranked in the top 10%. Two were in the top 10 compounds, four in the top 20, and six in the top 40 (Table 3 and Additional file 1: Table S3).

#### LigGrep-filtered *Sc*Hxk2p virtual screen

To show how LigGrep can improve the hit rate of the *Sc*Hxk2p VS, we filtered the docked poses of all library compounds to identify those that positioned a glucose-like (tetrahydro-2*H*-pyran) moiety near the location where glucose, the endogenous substrate, binds (Table 3, see “Implementation” for details) [74]. LigGrep filtered out 428 of the 1693 unique compounds in the virtual library. We ranked (by docking score) the remaining 1265 unique compounds with poses that matched our filter criteria, including 40 of the positive-control compounds. After LigGrep filtering, the number of positive controls in the top 10 highest-scoring compounds doubled to four. Six positive controls ranked in the top 20, and seven in the top 40 (Table 3 and Additional file 1: Table S3). The enrichment factors (Fig. 2) and areas under the pROC curve also improved (Additional file 1: Figure S1 and Table S4).

### LigGrep advantages and disadvantages

To illustrate the advantages and disadvantages of the LigGrep approach, we now consider in detail several of the docked poses from our *Hs*PARP1 VS.

#### Examples that illustrate LigGrep advantages

**Fig. 3:**
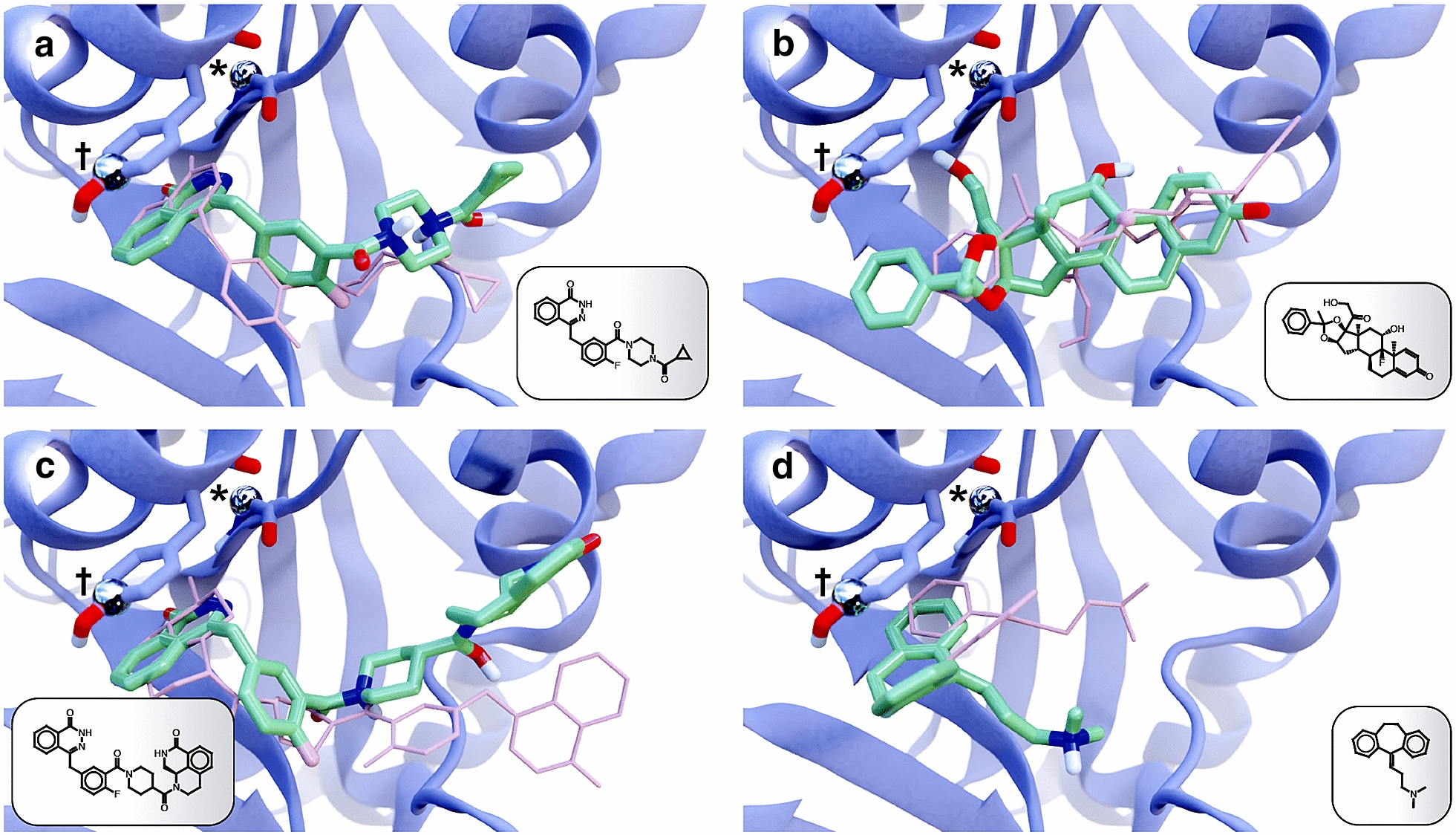
Example poses taken from a benchmark *Hs*PARP1 VS. Docked ligand poses are shown in green, and the *Hs*PARP1 receptor (PDB: 6BHV) is shown in blue. The atoms used to define the hydrogen-bond and $\pi$-$\pi$ LigGrep filters (metallic spheres) are labeled with an asterisk and dagger, respectively. **a** Olaparib, with a generally correct docked pose that passed all LigGrep filters. The crystallographic pose is shown in pink. **b** Tricinolone acetophenonide (NSC37641), a high-scoring decoy molecule, had a top-scoring docked pose did not pass LigGrep filters (in pink), but a lower-scoring pose that did (in green). **c** Compound 33, with an incorrect docked pose that nevertheless passed LigGrep filters. The crystallographic pose is shown in pink. **d** Amitriptyline, a true ligand that could not have passed LigGrep filters, regardless of pose accuracy. The crystallographic pose is shown in pink

LigGrep can improve hit rates by (1) eliminating compounds that are less likely to bind the target protein, and (2) allowing researchers to consider all poses (rather than only the top-scoring pose) when searching for potential ligands. The low-nanomolar *Hs*PARP1 inhibitor olaparib [28] illustrates the first advantage (Fig. 3a). The docked and crystallographic poses of olaparib were similar (RMSD: 2.7 Å; Fig. 3a in green and pink, respectively) [75]. Prior to LigGrep filtering, olaparib ranked third in our VS (−12.1 kcal/mol), behind a presumed decoy in second place. But none of the poses associated with the second-place compound passed the filters, so olaparib moved from third to second.

Tricinolone acetophenonide (NSC37641), a high-scoring decoy molecule, illustrates the second advantage of the LigGrep approach (Fig. 3b). The top-scoring pose of this compound (in pink) has a Vina docking score of −10.4 kcal/mol, but it does not position an oxygen or nitrogen atom close enough to the *Hs*PARP1 G863 residue, as required by one of our LigGrep filters. Had we manually inspected only the top-scoring pose associated with each ligand, we may therefore have discarded this compound, despite its impressive docking score (top $\sim$3% of all compounds). But LigGrep allowed us to identify a second, slightly lower-scoring pose (−10.2 kcal/mol) that did in fact position a compound hydroxyl group near the G863 residue. Of course, tricinolone acetophenonide is not known to inhibit *Hs*PARP1, and it was in fact included in the screen as a decoy (presumed inactive) compound. But this example nevertheless illustrates how LigGrep allows researchers to identify reasonable albeit lower-scoring docked poses, even when the top-scoring pose is implausible.

#### Examples that illustrate LigGrep disadvantages

By way of disadvantages, we note that (1) some incorrect poses nevertheless pass LigGrep filters, and (2) some true ligands may not contain the substructures required to pass, regardless of pose accuracy. “Compound 33,” a low-nanomolar benzo[*de*][1,7]naphthyridin-7(8*H*)-one inhibitor [37], illustrates the first disadvantage. Though it was the top-scoring compound in our VS (−13.4 kcal/mol) and passed our LigGrep filters, its pose is notably incorrect (12.1 Å RMSD from the crystallographic pose). The inhibitor coincidentally has similar substructures at both its ends: phthalazin-1(2*H*)-one and 3,4-dihydroisoquinolin-1(2*H*)-one, respectively. In the docked pose (Fig. 3c, green), the compound was flipped in the binding pocket relative to the crystallographic pose (Fig. 3c, pink), such that the phthalazin-1(2*H*)-one substructure satisfied the filters rather than the (correct) 3,4-dihydroisoquinolin-1(2*H*)-one substructure.

Amitriptyline provides an example of the second disadvantage of the LigGrep approach. This low-micromolar *Hs*PARP1 ligand ranked 229th out of 1561 compounds (−9.2 kcal/mol, 15th percentile) in our pre-LigGrep VS, and its docked and crystallographic poses were fairly similar (RMSD 3.8 Å, Fig. 3d, green and pink, respectively) [76]. But amitriptyline lacks a nitrogen/oxygen atom adjacent to an aromatic ring and so could not ever pass our LigGrep filters, regardless of its pose.

### Comparison with existing programs

Several powerful commercial docking programs also allow users to filter docked poses or to otherwise apply constraints during the docking process. LigGrep’s main advantage is that it can be applied to VS performed with free, open-source docking programs that often lack built-in pose filters. In contrast, commercial programs are often expensive and have restrictive licenses that impose substantial commercialization and intellectual-property restrictions. Furthermore, in some cases license eligibility is regularly re-evaluated, making long-term access uncertain. We here compare two commercial programs to LigGrep and describe how LigGrep can complement their native functionality.

#### Schrödinger Glide

Schrödinger’s Glide [77, 78] is a state-of-the-art commercial docking program that allows the user to apply constraints both during the docking process (such that they impact poses and scores) or after docking (as post-VS filters similar to LigGrep). Glide can account for (1) positional filters, which require a given docked-compound atom to occupy a user-defined spherical region; (2) excluded volumes, which require the compound to avoid defined regions of space; (3) nuclear Overhauser effect (NOE) constraints, which require a given protein/ligand atom-atom distance to fall within a user-provided range; and (4) hydrogen-bond/metal/metal-coordination constraints, which require the candidate ligand to form key interactions with receptor functional groups [79]. The positional and excluded-volume filters are notably similar to those that LigGrep implements.

We anticipate that most Glide users will prefer to use Glide’s built-in constraints, but it is certainly possible to apply LigGrep filters to Glide-docked poses as well. Schrödinger’s Maestro Suite can export protein receptor and small-molecule models as PDB and SDF files, respectively [78], formats that LigGrep can in turn accept as input.

#### OpenEye FRED

OpenEye’s FRED is another popular commercial docking program that includes both protein and custom filters [80, 81]. A FRED protein filter is satisfied when a docked compound is predicted to participate in a user-specified interaction (hydrogen bond, metal-chelator, contact, etc.) with a given protein atom. A custom filter is satisfied when a SMARTS-specified small-molecule substructure occupies a user-specified sphere. This last filter type in particular is very reminiscent of the LigGrep approach. As with Glide, FRED can also output docked poses in the SDF format, so LigGrep filters can be applied to FRED-docked poses as well.

## Conclusion

LigGrep allows researchers performing VS to improve hit rates by leveraging prior knowledge about key receptor/ligand interactions known to correlate with activity. Our results demonstrate that LigGrep can effectively filter out decoy molecules while retaining known ligands. In three separate test cases, LigGrep filtering improved hit rates over those obtained using AutoDock Vina alone. LigGrep will be a useful tool for the CADD community. We release it under the terms of the Apache License, Version 2.0. A copy is freely available at http://durrantlab.com/liggrep/.

## Supplementary information

**Additional file 1: Table S1.** Poly(ADP-ribose) polymerase 1 (PARP1) virtual screen, before and after applying two LigGrep filters (detailed results). **Table S2.** *H. sapiens* peptidyl-prolyl cis-trans isomerase NIMA-interacting 1 (*Hs*Pin1p) virtual screen, before and after applying a LigGrep filter (detailed results). **Table S3.** *S. cerevisiae* hexokinase-2 (*Sc*Hxk2p) virtual screen, before and after applying a LigGrep filter (detailed results). **Table S4.** AUROC and pAUROC values before and after LigGrep filtering. **Figure S1.** pROC curves describing our PARP1, *Hs*Pin1p, and *Sc*Hxk2p VS, before (blue) and after (orange) applying LigGrep filters.

## Data Availability

Project name: LigGrep 1.0.0. Project home page: http://durrantlab.com/liggrep. Operating systems: macOS, Linux, Windows. Programming language: Python 3. Other requirements: RDKit, NumPy, SciPy, Open Babel (optional). License: Apache License, Version 2.0. Restrictions to use by non-academics: None
