# Supplementary material for: LigGrep: a tool for filtering docked poses to improve virtual-screening hit rates
Source: J Cheminform. 2020 Nov 11;12:69. doi: 10.1186/s13321-020-00471-2 (PMC7656723; doi:10.1186/s13321-020-00471-2)
Supplement: Supplementary file 1 — Additional file 1: Table S1. Poly(ADP-ribose) polymerase 1 (PARP1) virtual screen, before and after applying two LigGrep filters (detailed results). Table S2. H. sapiens peptidyl-prolyl cis-trans isomerase NIMA-interacting 1 (HsPin1p) virtual screen, before and after applying a LigGrep filter (detailed results). Table S3. S. cerevisiae hexokinase-2 (ScHxk2p) virtual screen, before and after applying a LigGrep filter (detailed results). Table S4. AUROC and pAUROC values before and after LigGrep filtering. Figure S1. pROC curves describing our PARP1, HsPin1p, and ScHxk2p VS, before (blue) and after (orange) applying LigGrep filters. [file 13321_2020_471_MOESM1_ESM.pdf]

Table S1: Poly(ADP-ribose) polymerase 1 (PARP1) virtual screen, before and after applying two LigGrep filters. The virtual library included 1,561 unique molecules (46 known PARP1 ligands, represented by their PDB IDs, and 1,515 presumed decoys). Given that (1) Gypsum-DL generated up to two molecular variants (e.g., protonation states) for each molecule and (2) Vina produced up to 9 candidates poses per input file, there were 24,011 poses total. Only 1,126 unique molecules were associated with poses that passed our LigGrep filters, including 45/46 of the known PARP1 ligands. We note that the 6BHV structure captures its ligand in two distinct orientations. We used the crystallographic pose most similar to the docked pose to calculate the root-mean-square deviation (RMSD).

Ligand	Vina Score	RMSD	Vina Rank / Percentile	LigGrep Rank / Percentile
4HHY	-13.4	12.14	1 / 0.06%	1 / 0.09%
5DS3	-12.1	2.72	3 / 0.2%	2 / 0.2%
4HHZ	-12.0	3.38	5 / 0.3%	3 / 0.3%
6NRF	-11.8	3.37	6 / 0.3%	4 / 0.4%
6NRH	-11.5	2.97	9 / 0.6%	6 / 0.5%
6NRJ	-11.2	3.42	13 / 0.8%	7 / 0.6%
6NRI	-11.2	3.64	14 / 0.9%	8 / 0.7%
5KPP	-10.9	2.03	24 / 1.5%	14 / 1.2%
1UK0	-10.8	2.26	27 / 1.7%	16 / 1.4%
5WTC	-10.8	1.72	28 / 1.8%	17 / 1.5%
5KPO	-10.6	2.00	35 / 2.2%	19 / 1.7%
5KPN	-10.6	1.88	39 / 2.5%	21 / 1.9%
6NRG	-10.5	3.35	40 / 2.6%	22 / 2.0%
5A00	-10.5	6.95	45 / 2.9%	24 / 2.1%
6BHV	-10.4	2.64	48 / 3.1%	27 / 2.4%
5KPQ	-10.4	2.04	53 / 3.4%	30 / 2.7%
6GHK	-10.3	3.51	59 / 3.8%	33 / 2.9%
4PJT	-10.2	2.47	65 / 4.2%	38 / 3.4%
4UND	-10.2	2.04	66 / 4.2%	39 / 3.5%
4RV6	-10.1	5.14	80 / 5.1%	82 (score -9.7) / 7.3%
5WRQ	-10.0	2.34	87 / 5.6%	50 / 4.4%
1UK1	-10.0	2.05	95 / 6.1%	53 / 4.7%
5WRY	-9.9	2.03	101 / 6.5%	60 / 5.3%
4OQA	-9.9	3.01	100 / 6.4%	59 / 5.2%
3L3M	-9.9	0.71	103 / 6.6%	62 / 5.5%
4L6S	-9.8	2.26	114 / 7.3%	66 / 5.9%
4OQB	-9.8	1.62	119 / 7.6%	69 / 6.1%
4R6E	-9.6	5.36	153 / 9.8%	130 / 11.5%
4UXB	-9.2	0.85	222 / 14.2%	139 / 12.3%
5HA9	-9.2	3.75	229 / 14.7%	DID NOT CATCH
3L3L	-9.1	3.90	260 / 16.7%	163 / 14.5%
5XSR	-9.0	1.16	281 / 18.0%	178 / 15.8%
2RCW	-8.9	3.00	295 / 18.9%	186 / 16.5%
5WRZ	-8.9	1.41	298 / 19.1%	188 / 16.7%
5XSU	-8.9	0.91	314 / 20.1%	199 / 17.7%
1WOK	-8.9	1.76	319 / 20.4%	204 / 18.1%
4R5W	-8.8	7.43	333 / 21.3%	213 / 18.9%
5XST	-8.8	0.87	336 / 21.5%	214 / 19.0%
5WS0	-8.7	2.77	381 / 24.4%	242 / 21.5%
3GJW	-8.7	2.06	390 / 25.0%	251 / 22.3%
3GN7	-8.4	1.99	486 / 31.1%	329 / 29.2%
2RD6	-8.4	0.96	502 / 32.2%	344 / 30.6%
5WS1	-8.4	2.95	510 / 32.7%	350 / 31.1%
4ZZZ	-8.2	3.57	597 / 38.2%	400 / 35.5%
4OPX	-8.2	0.48	599 / 38.4%	402 / 35.7%
4GV7	-7.5	0.74	930 / 59.6%	675 / 59.9%

Table S2: *H. sapiens* peptidyl-prolyl cis-trans isomerase NIMA-interacting 1 (*Hs*Pin1p) virtual screen, before and after applying a LigGrep filter. The virtual library included 1,542 unique molecules (27 known *Hs*Pin1p ligands, represented by their PDB IDs, and 1,515 presumed decoys). Given that (1) Gypsum-DL generated up to two molecular variants (e.g., protonation states) for each molecule and (2) Vina produced up to 9 candidates poses per input file, there were 23,598 poses total. Only 513 unique molecules were associated with poses that passed our LigGrep filter, including 25/27 of the known *Hs*Pin1p ligands.

Ligand	Vina Score	RMSD	Vina Rank / Percentile	LigGrep Rank / Percentile
4TYO	-8.2	1.65	2 / 0.13%	1 / 0.19%
3KAF	-7.8	1.94	7 / 0.45%	3 / 0.58%
3IKG	-7.6	5.17	19 / 1.2%	9 / 1.8%
3JYJ	-7.5	5.93	24 / 1.6%	33 (score -7.2) / 6.4%
2XPB	-7.4	6.81	36 / 2.3%	15 / 2.9%
3KAH	-7.3	1.90	53 / 3.4%	24 / 4.7%
3KAI	-7.3	8.30	54 / 3.5%	25 / 4.9%
2XP8	-7.2	4.26	72 / 4.7%	32 / 6.2%
3TC5	-7.1	2.98	98 / 6.4%	42 / 8.2%
2XP6	-7.1	0.26	99 / 6.4%	43 / 8.4%
2XPA	-7.0	4.46	131 / 8.5%	54 / 10.5%
3IKD	-7.0	5.07	132 / 8.6%	55 / 10.7%
2XP9	-6.9	6.34	183 / 11.9%	68 / 13.2%
3KAD	-6.8	1.69	238 / 15.4%	82 / 15.9%
3KAG	-6.7	3.20	303 / 19.6%	100 / 19.5%
2XP7	-6.7	0.91	304 / 19.7%	101 / 19.7%
3TCZ	-6.7	4.85	305 / 19.8%	102 / 19.9%
3NTP	-6.7	3.94	306 / 19.8%	103 / 20.1%
2XP5	-6.6	0.96	370 / 24.0%	124 / 24.2%
2XP3	-6.6	2.39	371 / 24.1%	125 / 24.4%
3ODK	-6.6	1.74	372 / 24.1%	126 / 24.6%
3KAC	-6.5	1.16	443 / 28.7%	DID NOT CATCH
2XP4	-6.3	0.59	611 / 39.6%	214 / 41.7%
4TNS	-6.1	3.45	810 / 52.5%	DID NOT CATCH
3TDB	-5.9	8.63	966 / 62.7%	329 / 64.1%
3KCE	-5.6	2.05	1189 / 77.1%	410 / 79.9%
3KAB	-5.5	1.98	1247 / 80.9%	427 / 83.2%

Table S3: *S. cerevisiae* hexokinase-2 (*Sc*Hxk2p) virtual screen, before and after applying a LigGrep filter. The virtual library included 1,693 unique molecules (41 compounds known to bind hexokinases and glucokinases–though not necessarily *Sc*Hxk2p specifically–represented by their PDB and BindingDB IDs, as well as 1,652 presumed decoys). Given that (1) Gypsum-DL generated up to two molecular variants (e.g., protonation states) for each molecule and (2) Vina produced up to 9 candidate poses per input file, there were 30,474 poses total. Only 1,265 unique molecules were associated with poses that passed our LigGrep filter, including 40/41 of the known ligands. Given that none of the PDB ligands were co-crystallized with *Sc*Hxk2p, we do not include an RMSD analysis here.

Ligand	Vina Score	Vina Rank / Percentile	LigGrep Rank / Percentile
BDBM50169033	-9.2	6 / 0.4%	6 (score -8.9) / 0.5%
BDBM50169032	-9.1	8 / 0.5%	17 (score -8.7) / 1.3%
BDBM50169017	-9.0	18 / 1.1%	2 / 0.2%
BDBM50169031	-9.0	19 / 1.1%	3 / 0.2%
BDBM50169026	-8.9	24 / 1.4%	16 (score -8.7) / 1.3%
BDBM50169038	-8.9	25 / 1.5%	7 / 0.6%
BDBM50169034	-8.6	79 / 4.7%	77 (score -8.3) / 6.1%
BDBM50169023	-8.5	101 / 6.0%	40 / 3.2%
BDBM50169028	-8.5	102 / 6.0%	DID NOT CATCH
BDBM50169043	-8.5	103 / 6.1%	63 (score -8.4) / 5.0%
BDBM50103565	-8.4	138 / 8.2%	61 / 4.8%
BDBM50169040	-8.4	139 / 8.2%	62 / 4.9%
BDBM50169046	-8.2	216 / 12.8%	335 (score -7.6) / 26.5%
BDBM50169015	-8.1	268 / 15.8%	207 (score -7.9) / 16.4%
BDBM50169025	-8.1	269 / 15.9%	163 (score -8.0) / 12.9%
BDBM50169036	-8.1	270 / 16.0%	208 (score -7.9) / 16.4%
BDBM50169037	-8.1	271 / 16.0%	509 (score -7.3) / 40.2%
BDBM50169042	-8.1	272 / 16.1%	166 (score -8.0) / 13.1%
BDBM50169014	-8.0	326 / 19.3%	683 (score -7.0) / 54.0%
BDBM50169019	-8.0	327 / 19.3%	728 (score -6.9) / 57.5%
BDBM50169039	-8.0	328 / 19.4%	164 / 12.9%
BDBM50169041	-8.0	329 / 19.4%	165 / 13.0%
5BRH	-7.9	396 / 23.4%	295 (score -7.7) / 23.3%
5HG1	-7.9	397 / 23.4%	895 (score -6.6) / 70.8%
5BRF	-7.6	578 / 34.1%	727 (score -6.9) / 57.5%
BDBM50169018	-7.5	639 / 37.7%	508 (score -7.3) / 40.2%
BDBM50169013	-7.4	724 / 42.8%	565 (score -7.2) / 44.7%
BDBM85422	-7.4	725 / 42.8%	454 / 35.9%
BDBM50169020	-7.1	940 / 55.5%	629 / 49.7%
BDBM85424	-7.1	941 / 55.6%	630 / 49.8%
5BRE	-7.0	1012 / 59.8%	783 (score -6.8) / 61.9%
BDBM50169022	-7.0	1013 / 59.8%	684 / 54.1%
BDBM85421	-7.0	1014 / 59.9%	685 / 54.2%
BDBM85423	-6.9	1093 / 64.6%	943 (score -6.5) / 74.5%
BDBM85426	-6.9	1094 / 64.6%	785 (score -6.8) / 62.1%
BDBM85425	-6.8	1187 / 70.1%	784 / 61.9%
2YHX	-6.8	1188 / 70.2%	944 (score -6.5) / 74.6%
BDBM85420	-6.7	1272 / 75.1%	840 / 66.4%
5BRD	-6.7	1273 / 75.2%	896 (score -6.6) / 70.8%
2NZT	-5.7	1636 / 96.6%	1182 / 93.4%
3B8A	-5.5	1666 / 98.4%	1210 / 95.7%

1 ROC Metrics

LigGrep is best at enriching the set of top-scoring compounds for known ligands, but we also calculated the area under the receiver operating characteristic (ROC) curve (AUROC) of each virtual screening (VS) to evaluate overall performance [1]. The value of this area corresponds to the probability that a known ligand would rank better than a decoy if we were to randomly pick one of each from the set of docked compounds. We note that AUROC is a useful metric for evaluating a VS, but it gives equal consideration to the rankings of all docked compounds, from the best scoring to the worst. In practice, the top-scoring compounds (the ones that will be subsequently tested) are of primary interest.

To compensate for this shortcoming in part, we also calculated the area under the pROC curve for each VS. These curves give greater weight to top-scoring compounds. A pROC curve is derived from a ROC curve, but the negative logarithm (base 10) of the false-positive rate (FPR) is used rather than the FPR itself. Because one cannot take the logarithm of zero, the FPR of the top-ranking compound is set to $1/N$, where N is the number of decoy molecules. pAUROC lacks the intuitive interpretation of AUROC, but it still serves as a useful metric for comparing different VS methodologies.

To calculate the areas under all curves (ROC and pROC), we used SciPy [2] to (1) find a B-spline interpola-tion of the data [3], (2) evaluate the B-spline at 1,000 evenly spaced points, and (3) calculate the area by applying fixed-order Gaussian quadrature to those evaluations [4, 5]. To determine whether LigGrep can improve VS performance, we compared the AUROC and pAUROC of each VS before and after filtering docked poses using LigGrep.

1.1 PARP1

Prior to LigGrep filtering, the AUROC and pAUROC of the PARP1 Vina screen were 0.89 and 1.37, respectively. After filtering, the AUROC metric improved to 0.90 (Table S4). This improvement was only minor because the PARP1 VS was already very predictive, even before we applied LigGrep. But the pAUROC metric, which emphasizes the portion of the ranked compound list comprised of high-ranking compounds, increased to 1.54, showing that LigGrep was particularly effective at enriching the set of arguably most promising compounds (Table S4 and Figure S1).

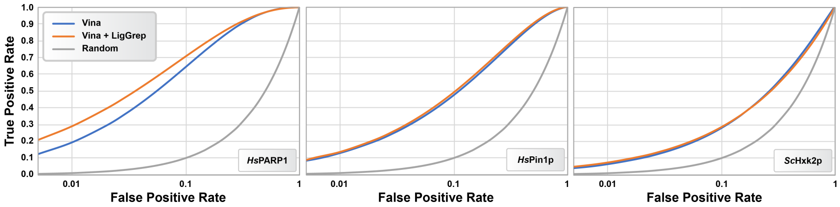

Figure S1: pROC curves describing our PARP1, *Hs*Pin1p, and *Sc*Hxk2p VS, before (blue) and after (orange) applying LigGrep filters. The theoretical curve that would be obtained had the compounds been ordered at random (without regard for docking score) is shown in grey. Note that the x axis is logarithmic, which distinguishes this plot from a standard ROC curve. The left-most point (corresponding to the top-ranked ligand) is omitted because one cannot take the logarithm of 0.

Table S4: AUROC and pAUROC values before and after LigGrep filtering.

Protein	Screen Type	AUROC	pAUROC
PARP1	Vina only	0.89	1.37
PARP1	Vina + LigGrep	0.90	1.54
*Hs*Pin1p	Vina only	0.81	1.11
*Hs*Pin1p	Vina + LigGrep	0.82	1.12
*Sc*Hxk2p	Vina only	0.66	0.76
*Sc*Hxk2p	Vina + LigGrep	0.65	0.77

1.2 *Hs*Pin1p

Prior to LigGrep filtering, the AUROC and pAUROC of the *Hs*Pin1p Vina screen were 0.81 and 1.11, respectively. After filtering, the AUROC and pAUROC metrics improved slightly to 0.82 and 1.12, respectively (Table S4), further confirming that LigGrep was effective (Table S4 and Figure S1).

1.3 *Sc*Hxk2p

As expected, the AUROC and pAUROC of the *Sc*Hxk2p Vina screen (0.66 and 0.76, respectively) were much lower than those of the other benchmark VS (Table S4). After LigGrep filtering, the AUROC metric decreased slightly to 0.65, though the pAUROC metric increased to 0.77 (Table S4). In this challenging case, LigGrep thus managed to improve the hit rate among the very top-scoring compounds, even though it had only minimal impact on the performance of the whole screen (Table S4 and Figure S1).

References

(1) Eng, J. ROC analysis: web-based calculator for ROC curves, 2020.
(2) Jones, E.; Oliphant, T.; Peterson, P., et al. SciPy: Open Source Scientific Tools for Python, Computer Program, 2001.
(3) Dierckx, P., *Curve and surface fitting with splines*; Clarendon: Oxford, 1993.
(4) Gauss, C. F., *Methodus nova integralium valores per approximationem inveniendi*; apvd Henricvm Dieterich: 1815.
(5) Jacobi, C. G. J. *Journal für die reine und angewandte Mathematik* **1826**, *1826*, 301–308.
